# CIPK11 Regulates Plant Adaptation to Nitrate Availability in Arabidopsis

**DOI:** 10.3390/plants15172723

**Published:** 2026-09-05

**Authors:** Haijing Wang, Tongtong Liu, Hongzhi Xu, Huien Wang, Xiubin Chen, Juanyong Liu, Li Liu, Xinhua Song, Xiuli Han

**Affiliations:** 1 School of Life Sciences and Medicine, Shandong University of Technology, Zibo 255049, China; 19862703985@163.com (H.W.); 19553302807@163.com (H.W.); 17861026559@163.com (X.C.); liuli6687@163.com (L.L.); 2 Analysis and Testing Center, Shandong University of Technology, Zibo 255049, China

**Keywords:** nitrate availability, CIPK11, PM H^+^-ATPase

## Abstract

Nitrate (NO_3_^−^) is an essential nitrogen source and an important signaling molecule that regulates plant growth and development in response to nitrogen availability. Although several CBL-interacting protein kinases (CIPKs) have been implicated in nitrate signaling, the role of CIPK11 (PKS5) in nitrate availability responses remains unknown. Here, we investigated the function of CIPK11 using Arabidopsis mutants under nitrate-deficient and nitrate-resupply conditions by analyzing growth phenotypes, nitrate and protein accumulation, nitrate-responsive gene expression, extracellular H^+^ dynamics, and responses to the plasma membrane (PM) H^+^-ATPase inhibitor protonstatin-1 (PS-1). *CIPK11* expression was downregulated under nitrate deficiency and rapidly induced after nitrate resupply. The loss-of-function mutant *pks5-1* exhibited enhanced growth, increased nitrate and protein accumulation, and stronger induction of nitrate-responsive genes (*NRT2.1*, *NRT2.4*, *NIA1*, and *NIA2*), whereas the gain-of-function mutants *pks5-3* and *pks5-4* showed opposite phenotypes. Altered extracellular H^+^ dynamics and PS-1 sensitivity further suggested that PM H^+^-ATPase activity contributes to CIPK11-mediated nitrate responses. Collectively, our findings identify CIPK11 as a negative regulator of plant adaptation to nitrate availability and suggest that CIPK11 modulates nitrate acquisition and assimilation, at least partly through regulation of PM H^+^-ATPase activity.

## 1. Introduction

Nitrogen (N) is an essential element for plant growth and development, constituting 1–5% of the total dry matter in plants and occurring in various biomolecules, including proteins, nucleic acids, and chlorophyll [1]. In agricultural systems, insufficient N supply can severely constrain crop productivity, whereas excessive N fertilization causes environmental problems such as eutrophication [2]. Plants have evolved sophisticated mechanisms to adapt to fluctuations in N availability [3], and improving N use efficiency (NUE) by elucidating the molecular mechanisms of N signaling represents an effective strategy to address these challenges [2,4].

Nitrate, the primary inorganic N source for plants, functions not only as a vital nutrient but also as an important signaling molecule in response to fluctuations in N availability [5]. Nitrate uptake is mediated by nitrate transporters (NRTs), which are classified as high-affinity transporters (HATs) and low-affinity transporters (LATs) [6]. HATs operate under low-nitrate conditions, typically below 1 mM, with NRT2.1, NRT2.4, and NRT2.5 playing central roles in root nitrate uptake in Arabidopsis [6]. LATs function at higher nitrate concentrations and are primarily represented by members of the NRT1 family, with the exception of NRT1.1 (also named as CHL1/NPF6.3), a dual-affinity transporter whose activity is regulated by phosphorylation [1,6]. After uptake, NO_3_^−^ is sequentially reduced to NO_2_^−^ by nitrate reductase (NIA) and to NH_4_^+^ by nitrite reductase (NiR), before being incorporated into amino acids via the glutamine synthetase (GS)/glutamine-2-oxoglutarate aminotransferase (GOGAT) cycle [4,7].

Plant responses to nitrate availability involve both short-term and long-term mechanisms. The short-term primary nitrate response is characterized by rapid induction of nitrate-responsive genes, including *NRTs*, *NIAs*, and *NiRs*, whereas long-term responses affect overall growth and development, particularly root system architecture (RSA) [2,8]. RSA adapts to nitrate availability by developing a steeper root angle, which facilitates N foraging from subsoil layers under N-deficient conditions [8]. Moreover, root growth is regulated in a nitrate dose-dependent manner: moderate nitrate supply (between 550 µM and 275 µM N supply) promotes the elongation of both primary and lateral roots, whereas severe N deficiency (≤100 µM N supply) suppresses root growth [8,9,10].

Ca^2+^ signaling plays a critical role in regulating nitrate-responsive genes, with the Ca^2+^-calcineurin B-like (CBL)-CBL-interacting protein kinase (CIPK) module serving as an important signaling pathway [11]. The Ca^2+^-CBL-CIPK network constitutes a versatile nutrient signaling system, with 10 CBLs and 26 CIPKs identified in Arabidopsis [11,12]. This module regulates both primary nitrate responses and long-term nitrate-dependent growth by modulating the phosphorylation of numerous proteins, including membrane-localized nitrate transporters [12,13]. Specific components, such as NRT1.1, function as nitrate sensors, exhibiting high-affinity nitrate uptake when threonine 101 (T101) is phosphorylated by the CBL9/CBL1-CIPK23 complex, and low affinity when dephosphorylated [11,14]. CIPK8 in Arabidopsis positively regulates the primary nitrate responses through the upregulation of *NRTs*, *NIAs*, and *NiR* [15]. CIPK1 plays a role in induction of N-starvation responsive genes under N deficiency, and the maintenance of plant growth in Arabidopsis [16]. In rice, OsCBL1 also positively regulates nitrate signaling and nitrate-mediated growth under conditions of limited nitrate supply [17,18]. Similarly, CBL7 in Arabidopsis is induced under low-nitrate conditions and promotes plant responses to nitrate, both in the primary nitrate response through induction of *NRT2.4* and *NRT2.5* and in long-term nitrate signaling by maintaining root growth [19]. Despite these insights, the coordinated roles of other CBLs and CIPKs in decoding Ca^2+^ signals remain largely unresolved.

PM H^+^-ATPases play fundamental roles in plant growth and environmental adaptation by establishing the transmembrane proton electrochemical gradient that drives numerous secondary transport processes, including nitrate uptake, which drive NO_3_^−^/H^+^ co-transport via NRT1 and NRT2 transporters [20,21]. Arabidopsis contains 11 PM H^+^-ATPase isoforms (AHA1–AHA11), among which AHA2 is predominantly expressed in roots and contributes to root architecture remodeling in response to nitrogen availability [22]. Post-translational modification, particularly phosphorylation of the C-terminal autoinhibitory domain, represents a major regulatory mechanism controlling PM H^+^-ATPase activity [23]. Phosphorylation at Thr947 and Thr881 enhances PM H^+^-ATPase activation, whereas phosphorylation at Ser931, Ser899, and Thr924 negatively regulates its activity [20,23]. The Ca^2+^-CBL-CIPK signaling module has been implicated in the regulation of PM H^+^-ATPase activity. In particular, phosphorylation levels of Thr947 and Thr881 in AHA2 were reduced in the *cbl1/4/5/8/9* quintuple mutant compared with the wild type, suggesting coordinated regulation of H^+^ and NO_3_^−^ fluxes in response to nitrate availability [12]. Indeed, higher levels and enhanced activity of PM H^+^-ATPase are closely associated with increased efficiency of nitrate uptake and assimilation in rice seedlings [24,25]. Nevertheless, the precise mechanisms underlying how PM H^+^-ATPase activity responds to fluctuations in nitrate availability remain largely unclear.

CIPK11 has been reported to phosphorylate AHA2 at Ser931 under nitrate-sufficient, non-stress conditions, thereby negatively regulating PM H^+^-ATPase activity to maintain proton pump activity at a relatively low level under favorable growth conditions [26]. However, under alkaline or salt stress conditions, Ca^2+^-activated 14-3-3 proteins inhibit CIPK11 activity and promote binding to phosphorylated Thr947 of AHA2, leading to activation of PM H^+^-ATPase [21,26]. Given that CIPK11 acts as a negative regulator of PM H^+^-ATPase under nitrate-sufficient conditions, the role of CIPK11-mediated PM H^+^-ATPase regulation in plant responses to nitrate fluctuations remains unclear.

In this study, we investigated the role of CIPK11 in plant responses to nitrate fluctuations and elucidated its underlying regulatory mechanism through modulation of PM H^+^-ATPase activity.

## 2. Results

### 2.1. CIPK11 Transcription Responds Dynamically to Nitrate Availability

**Figure 1:**
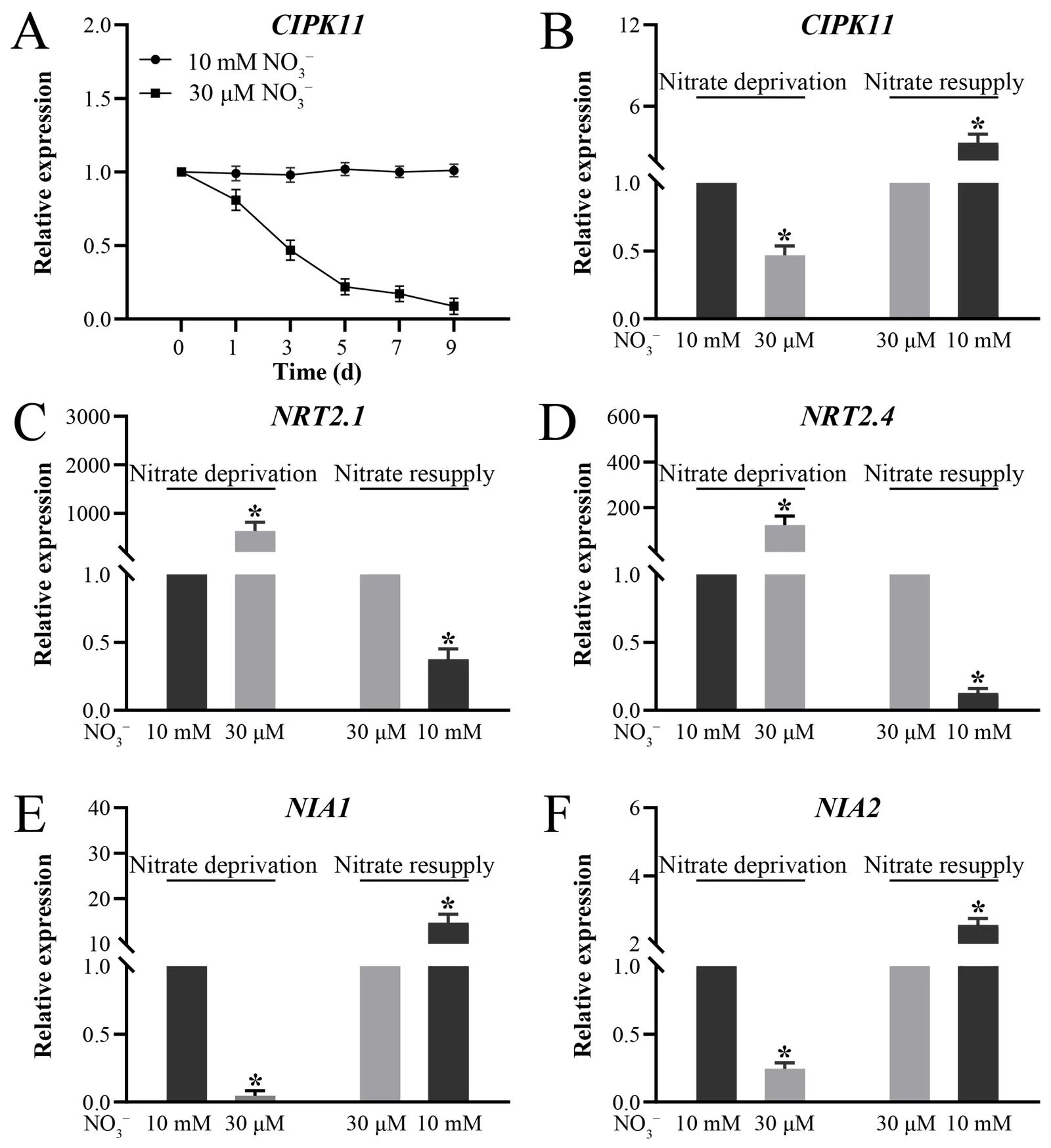
*CIPK11* expression responds dynamically to nitrate availability. (**A**) Relative expression of *CIPK11* during nitrate deprivation. 5-day-old *Arabidopsis thaliana* Col-0 seedlings grown on MS medium containing sufficient nitrate (10 mM NO_3_^−^) were transferred to either nitrate-sufficient (10 mM NO_3_^−^) or nitrate-deficient (30 μM NO_3_^−^) medium. Seedlings were harvested at the indicated time points for RNA extraction and qRT-PCR analysis. (**B**–**F**) Relative expression levels of *CIPK11*, *NRT2.1*, *NRT2.4*, *NIA1*, and *NIA2* under nitrate deprivation and subsequent nitrate resupply. 5-day-old Col-0 seedlings grown on MS medium containing 10 mM NO_3_^−^ were transferred to nitrate-deficient medium (30 μM NO_3_^−^) for 3 days. A subset of seedlings was harvested for RNA extraction and gene expression analysis, whereas the remaining seedlings were either transferred to nitrate-sufficient medium (10 mM NO_3_^−^) or transferred to nitrate-deficient medium (30 μM NO_3_^−^) for 3 h before sample collection. *ACTIN* was used as the internal control. Data are presented as the mean ± SD of three biological replicates (*n* = 3). Statistical significance was determined using Student’s *t*-test for comparisons between nitrate-sufficient (10 mM NO_3_^−^) and nitrate-deficient (30 μM NO_3_^−^) conditions, and between nitrate-deficient (30 μM NO_3_^−^) and nitrate-resupplied (10 mM NO_3_^−^) conditions; Asterisks indicate significant differences (*p* ≤ 0.05).

Although CIPK11 has been implicated in various stress responses, including salt stress [21], drought stress [27], cadmium toxicity [28], and iron acquisition [29], its role in nitrate response has not yet been investigated. To address this question, the temporal expression pattern of *CIPK11* was examined in seedlings exposed to nitrate-deficient conditions (30 μM NO_3_^−^, representing the residual background nitrate derived from other medium components during preparation). *CIPK11* transcript levels decreased progressively during nitrate deprivation (Figure 1A,B), indicating a time-dependent repression of *CIPK11* expression under nitrate-deficient conditions. In parallel, the expression of the high-affinity nitrate transporter genes *NRT2.1* and *NRT2.4* was markedly induced (Figure 1C,D), whereas the nitrate assimilation genes *NIA1* and *NIA2* were significantly repressed (Figure 1E,F), consistent with the transcriptional reprogramming typically associated with nitrate deficiency.

To further evaluate the responsiveness of CIPK11 to changes in nitrate availability, nitrate-starved seedlings were resupplied with sufficient nitrate (10 mM NO_3_^−^). Nitrate resupply rapidly increased *CIPK11* expression, accompanied by the downregulation of *NRT2.1* and *NRT2.4* and the induction of *NIA1* and *NIA2* (Figure 1B–F). However, the expression patterns of these genes did not fully return to the levels observed under continuous nitrate provision after 3 h of nitrate resupply. Specifically, *CIPK11* expression remained lower (Figure S1A), whereas *NRT2.1* and *NRT2.4* expression remained higher (Figure S1B,C), and *NIA1* and *NIA2* expression remained lower (Figure S1D,E) than those under continuous nitrate provision. Together, these results demonstrate that *CIPK11* expression responds dynamically to nitrate availability, suggesting a potential role for CIPK11 in regulating plant adaptation to fluctuating nitrate conditions.

### 2.2. CIPK11 Negatively Regulates Plant Growth Under Nitrate-Deficient Conditions

**Figure 2:**
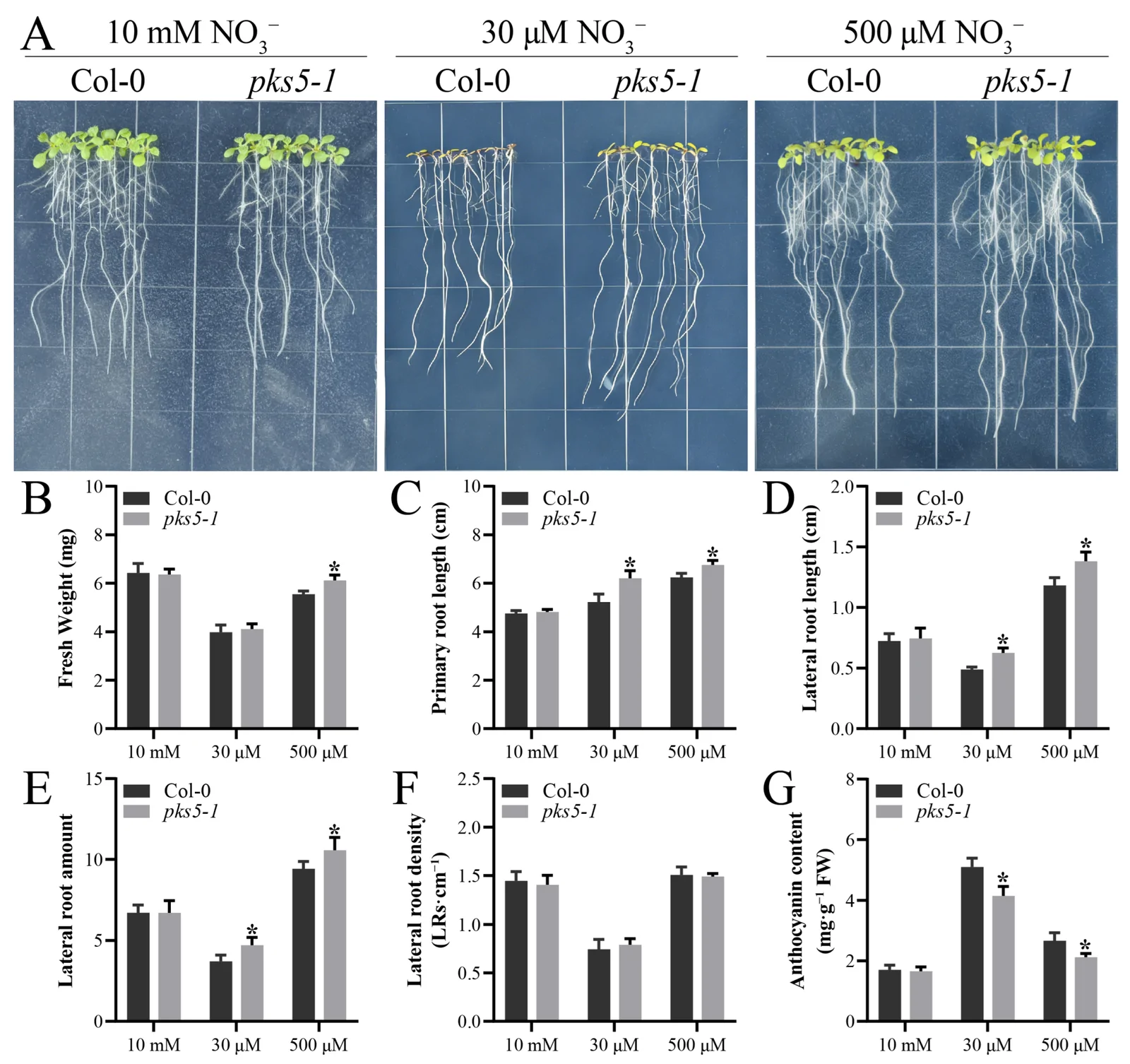
Impaired CIPK11 function enhances plant growth under nitrate-deficient conditions. (**A**) Growth phenotypes of Col-0 and *pks5-1* under nitrate-deficient conditions. 5-day-old seedlings grown vertically on MS medium containing 10 mM NO_3_^−^ were transferred to MS medium containing 10 mM, 30 μM, or 500 μM NO_3_^−^. Representative images were taken after 4 days of treatment. (**B**) Fresh weight of seedlings shown in (**A**). (**C**) Primary root length of seedlings shown in (**A**). (**D**) Lateral root length of seedlings shown in (**A**). (**E**) Lateral root amount of seedlings shown in (**A**). (**F**) Lateral root density of seedlings shown in (**A**). (**G**) Anthocyanin content of seedlings shown in (**A**). Data are presented as the mean ± SD of three biological replicates (*n* = 3). Statistical significance was determined using Student’s *t*-test for comparisons between Col-0 and *pks5-1* under the same treatment conditions; Asterisks indicate significant differences (*p* ≤ 0.05).

To investigate the role of CIPK11 in plant adaptation to nitrate deficiency, the growth phenotype of the CIPK11 loss-of-function mutant *pks5-1* was examined under different nitrate conditions. In this study, 30 μM NO_3_^−^ was used to represent severe nitrate deficiency, 500 μM NO_3_^−^ was used to represent moderate nitrate limitation, and 10 mM NO_3_^−^ was used as the nitrate-sufficient condition. These nitrate concentrations were selected to evaluate the effects of CIPK11 across a gradient of nitrate availability. Under nitrate-sufficient conditions (10 mM NO_3_^−^), *pks5-1* exhibited no obvious growth differences compared with the wild type (Col-0). However, under nitrate-deficient conditions (30 μM and 500 μM NO_3_^−^), *pks5-1* seedlings displayed enhanced growth relative to Col-0 (Figure 2A). Consistently, fresh weight was significantly increased in *pks5-1* under 500 μM nitrate, whereas the difference was less pronounced under 30 μM nitrate (Figure 2B), likely reflecting the severe growth restriction imposed by extreme nitrate limitation.

Given the critical role of root system architecture in plant adaptation to low nitrate availability, root growth traits were further analyzed. Primary root length, lateral root length, and lateral root amount were all significantly increased in *pks5-1* under nitrate-deficient conditions (Figure 2C–E), whereas lateral root density was not significantly affected (Figure 2F). In addition, anthocyanin accumulation, a characteristic physiological response to nitrate deficiency, was reduced in *pks5-1* seedlings at both nitrate concentrations (Figure 2G). These results suggest that CIPK11 contributes to the regulation of plant growth and nitrate deficiency responses, with loss of CIPK11 function being associated with enhanced growth and reduced nitrate deficiency-induced stress responses.

**Figure 3:**
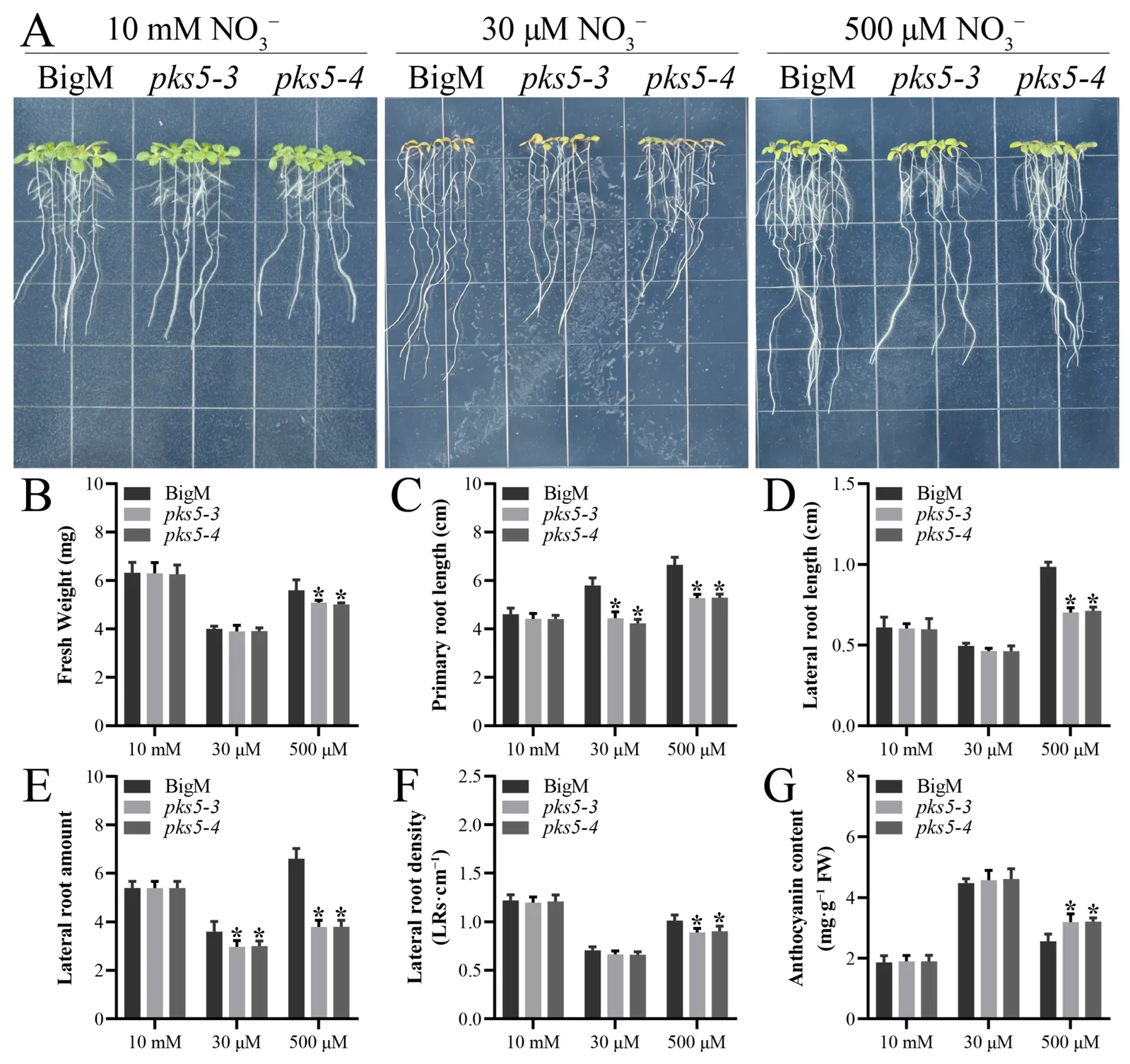
Enhanced CIPK11 function reduces plant growth under nitrate-deficient conditions. (**A**) Growth phenotypes of BigM, *pks5-3*, and *pks5-4* under nitrate-deficient conditions. Seedlings were treated as described in Figure 2A. (**B**) Fresh weight of seedlings shown in (**A**). (**C**) Primary root length of seedlings shown in (**A**). (**D**) Lateral root length of seedlings shown in (**A**). (**E**) Lateral root amount of seedlings shown in (**A**). (**F**) Lateral root density of seedlings shown in (**A**). (**G**) Anthocyanin content of seedlings shown in (**A**). Data are presented as the mean ± SD of three biological replicates (*n* = 3). Statistical significance was determined using Student’s *t*-test for comparisons of BigM with *pks5-3* and *pks5-4* under the same treatment conditions; Asterisks indicate significant differences (*p* ≤ 0.05).

To further verify the role of CIPK11, two gain-of-function mutants, *pks5-3* and *pks5-4*, were analyzed. In contrast to *pks5-1*, both gain-of-function mutants exhibited impaired growth under nitrate-deficient conditions (30 μM and 500 μM NO_3_^−^), as evidenced by reduced fresh weight and shorter primary and lateral roots (Figure 3A–D). Moreover, lateral root amount and density were significantly decreased in *pks5-3* and *pks5-4* under 500 μM NO_3_^−^ conditions (Figure 3E,F). Consistent with their growth inhibition, anthocyanin accumulation was significantly increased in both mutants compared with the corresponding control plants under 500 μM nitrate (Figure 3G). Together, these findings indicate that CIPK11 negatively regulates plant adaptation to nitrate-deficient conditions.

### 2.3. CIPK11 Regulates the Expression of Nitrate-Responsive Genes

Under nitrate-deficient conditions, high-affinity nitrate transporter genes, such as *NRT2.1* and *NRT2.4*, are typically upregulated, whereas nitrate assimilation genes, including *NIA1* and *NIA2*, are repressed [19]. To determine whether CIPK11 participates in the transcriptional regulation of these nitrate-responsive genes, their expression was examined in CIPK11 loss- and gain-of-function mutants.

**Figure 4:**
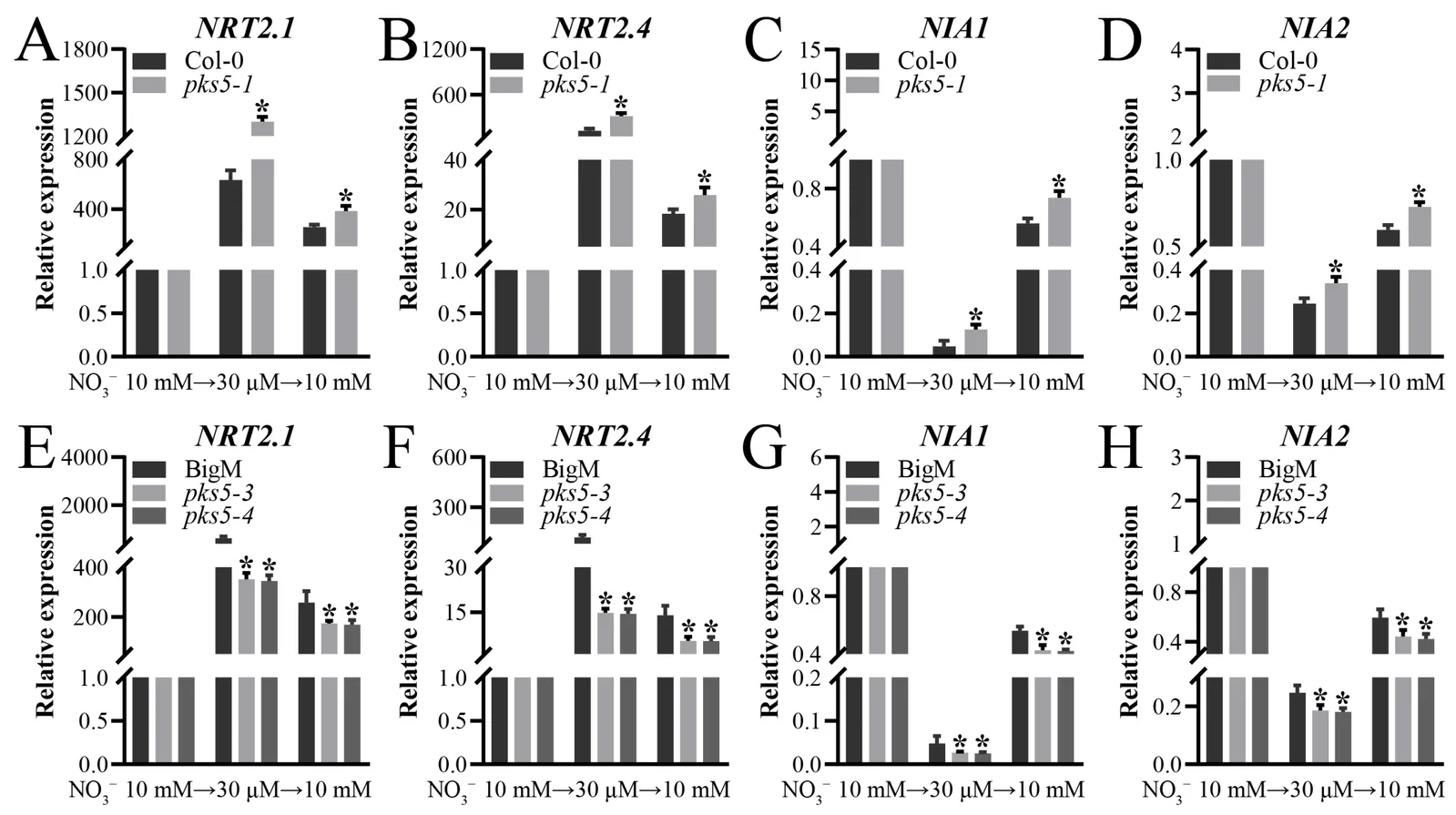
CIPK11 regulates the expression of nitrate-responsive genes in response to changes in nitrate availability. (**A**–**D**) Relative expression levels of *NRT2.1*, *NRT2.4*, *NIA1*, and *NIA2* in the CIPK11 loss-of-function mutant *pks5-1* during nitrate deprivation and subsequent nitrate resupply. (**E**–**H**) Relative expression levels of *NRT2.1*, *NRT2.4*, *NIA1*, and *NIA2* in the CIPK11 gain-of-function mutants *pks5-3* and *pks5-4* during nitrate deprivation and subsequent nitrate resupply. 5-day-old seedlings grown on MS medium containing 10 mM NO_3_^−^ were transferred to nitrate-deficient medium (30 μM NO_3_^−^) for 3 days. A subset of seedlings was harvested for RNA extraction and gene expression analysis, whereas the remaining seedlings were either transferred to nitrate-sufficient medium (10 mM NO_3_^−^) or transferred to nitrate-deficient medium (30 μM NO_3_^−^) for 3 h before sample collection for qRT-PCR analysis. *ACTIN* was used as the internal control. Data are presented as the mean ± SD of three biological replicates (*n* = 3). Statistical significance was determined using Student’s *t*-test for comparisons between Col-0 and *pks5-1*, and between BigM and *pks5-3* or *pks5-4* under the same treatment conditions; Asterisks indicate significant differences (*p* ≤ 0.05).

In the loss-of-function mutant *pks5-1*, nitrate deficiency induced the expression of *NRT2.1* and *NRT2.4* in a manner similar to that observed in Col-0; however, the magnitude of induction was significantly greater in *pks5-1* (Figure 4A,B). Similarly, although *NIA1* and *NIA2* were downregulated in both genotypes under nitrate-deficient conditions, the extent of repression was reduced in *pks5-1* (Figure 4C,D). In contrast, the gain-of-function mutants *pks5-3* and *pks5-4* exhibited weaker induction of *NRT2.1* and *NRT2.4* and stronger repression of *NIA1* and *NIA2* compared with the corresponding control plants (Figure 4E–H). These results indicate that CIPK11 negatively regulates the induction of nitrate transporter genes while promoting the repression of nitrate assimilation genes under nitrate-deficient conditions.

The results presented above showed that nitrate resupply following nitrate deprivation suppresses the expression of high-affinity nitrate transporter genes while inducing nitrate assimilation genes (Figure 1C–F). Because *CIPK11* expression was also rapidly induced upon nitrate resupply, we hypothesized that CIPK11 may participate in the transcriptional regulation of nitrate-responsive genes. To test this hypothesis, we analyzed the expression of representative nitrate transporter and assimilation genes following nitrate resupply. After 3 h of nitrate treatment, *NRT2.1* and *NRT2.4* expression decreased markedly in all genotypes; however, this repression was attenuated in *pks5-1* and enhanced in *pks5-3* and *pks5-4* relative to their respective controls (Figure 4A,B,E,F). In contrast, *NIA1* and *NIA2* were induced in all genotypes, with stronger induction observed in *pks5-1* and weaker induction in the gain-of-function mutants (Figure 4C,D,G,H).

Taken together, these findings demonstrate that CIPK11 modulates nitrate-responsive transcriptional reprogramming, thereby potentially limiting nitrate uptake and assimilation under fluctuating nitrate conditions.

### 2.4. CIPK11 Regulates Nitrate and Protein Accumulation in Response to Changes in Nitrate Availability

Because CIPK11 influences the expression of genes involved in nitrate uptake and assimilation and affects plant growth under nitrate-deficient conditions, we next examined whether nitrate accumulation and nitrate-derived protein synthesis are also regulated by CIPK11.

**Figure 5:**
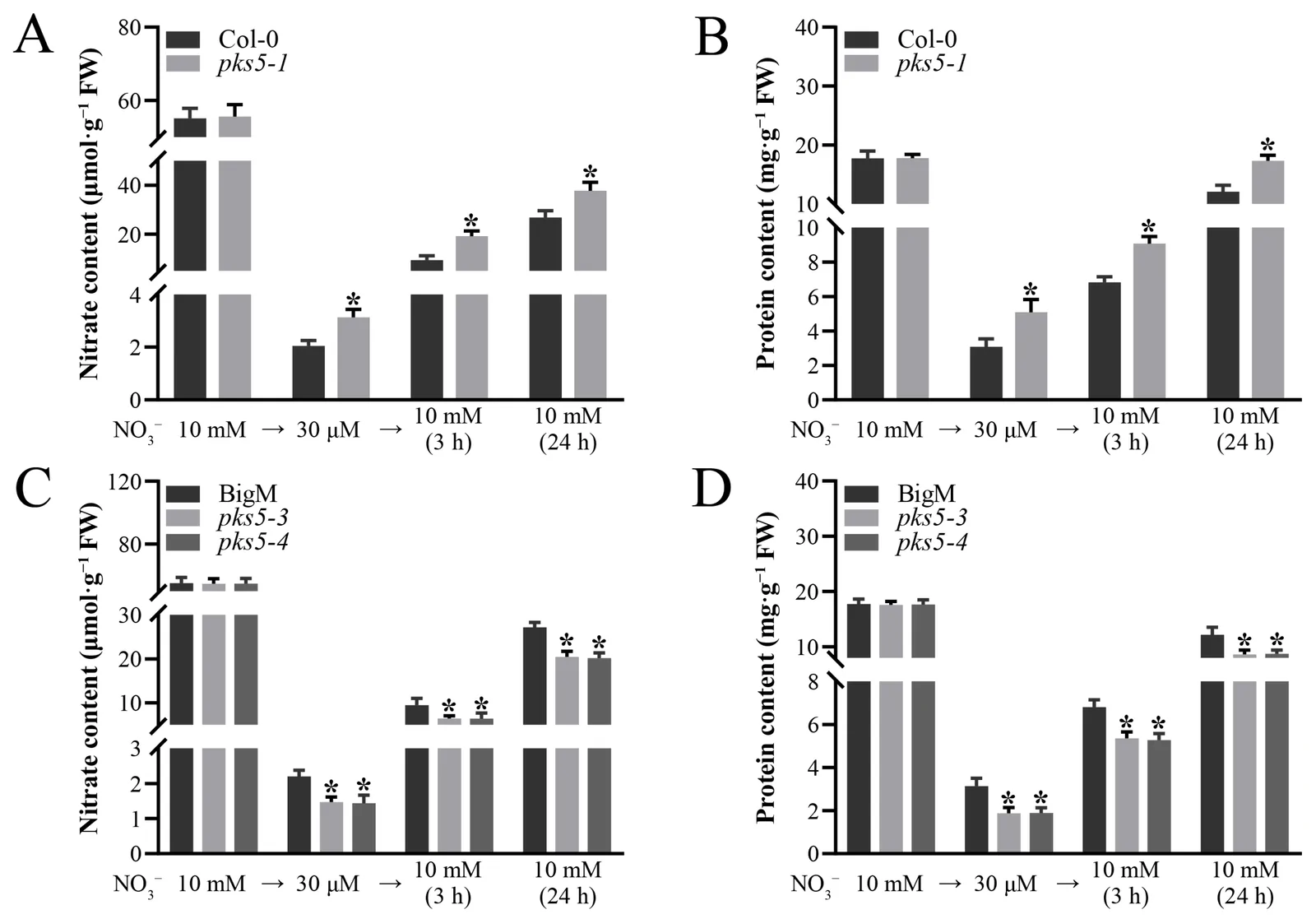
CIPK11 regulates nitrate and protein accumulation in response to changes in nitrate availability. (**A**) Nitrate content in Col-0 and *pks5-1* seedlings during nitrate deprivation and following nitrate resupply. (**B**) Protein content in Col-0 and *pks5-1* seedlings during nitrate deprivation and following nitrate resupply. (**C**) Nitrate content in BigM, *pks5-3*, and *pks5-4* seedlings during nitrate deprivation and following nitrate resupply. (**D**) Protein content in BigM, *pks5-3*, and *pks5-4* seedlings during nitrate deprivation and following nitrate resupply. 5-day-old seedlings grown on MS medium containing sufficient nitrate (10 mM NO_3_^−^) were transferred to nitrate-deficient medium (30 μM NO_3_^−^) for 3 days. For nitrate-resupply treatment, seedlings were subsequently transferred to nitrate-sufficient medium (10 mM NO_3_^−^) and harvested at the indicated time points for nitrate and protein content analyses. FW, fresh weight. Data are presented as the mean ± SD of three biological replicates (*n* = 3). Statistical significance was determined using Student’s *t*-test for comparisons between Col-0 and *pks5-1*, and between BigM and *pks5-3* or *pks5-4* under the same treatment condition; Asterisks indicate significant differences (*p* ≤ 0.05).

The nitrate and protein content analyses revealed that the loss-of-function mutant *pks5-1* accumulated significantly higher levels of nitrate and protein than Col-0 under both severe nitrate-deficient conditions (30 μM NO_3_^−^; Figure 5A,B) and moderate nitrate-deficient conditions (500 μM NO_3_^−^; Figure S2A,B).

In contrast, the gain-of-function mutants *pks5-3* and *pks5-4* exhibited lower nitrate and protein contents than the corresponding control line, BigM under both severe and moderate nitrate-deficient conditions (Figure 5C,D and Figure S2C,D). These findings indicate that CIPK11 negatively affects nitrate accumulation and influences nitrogen assimilation under nitrate-deficient conditions.

To further investigate the role of CIPK11 during recovery from nitrate deficiency, seedlings were resupplied with sufficient nitrate, and nitrate and protein contents were measured after 3 h and 24 h of treatment. Nitrate resupply resulted in progressive increases in both nitrate and protein contents in all genotypes (Figure 5A–D). Notably, *pks5-1* exhibited higher nitrate and protein contents than Col-0 (Figure 5A,B), whereas *pks5-3* and *pks5-4* displayed lower levels than BigM at both time points (Figure 5C,D). To further evaluate the changes in protein accumulation during nitrate recovery, net protein accumulation was calculated as the difference between protein content after nitrate resupply and that before resupply. Consistent with the total protein measurements, *pks5-1* showed higher net protein accumulation after 24 h of nitrate resupply, whereas *pks5-3* and *pks5-4* exhibited reduced net protein accumulation compared with BigM (Figure S3A,B).

Taken together, these results demonstrate that CIPK11 negatively regulates nitrate accumulation and nitrate-derived protein production under both nitrate-deficient and nitrate-resupply conditions, consistent with its role in repressing nitrate-responsive gene expression and limiting plant growth under low-nitrate conditions.

### 2.5. Potential Involvent of PM H^+^-ATPase in CIPK11-Mediated Nitrate Responses

PM H^+^-ATPases play essential roles in nutrient acquisition, including nitrate uptake, by generating the proton motive force required for NO_3_^−^/H^+^ co-transport [20]. Therefore, we investigated whether PM H^+^-ATPases participate in CIPK11-mediated nitrate responses.

**Figure 6:**
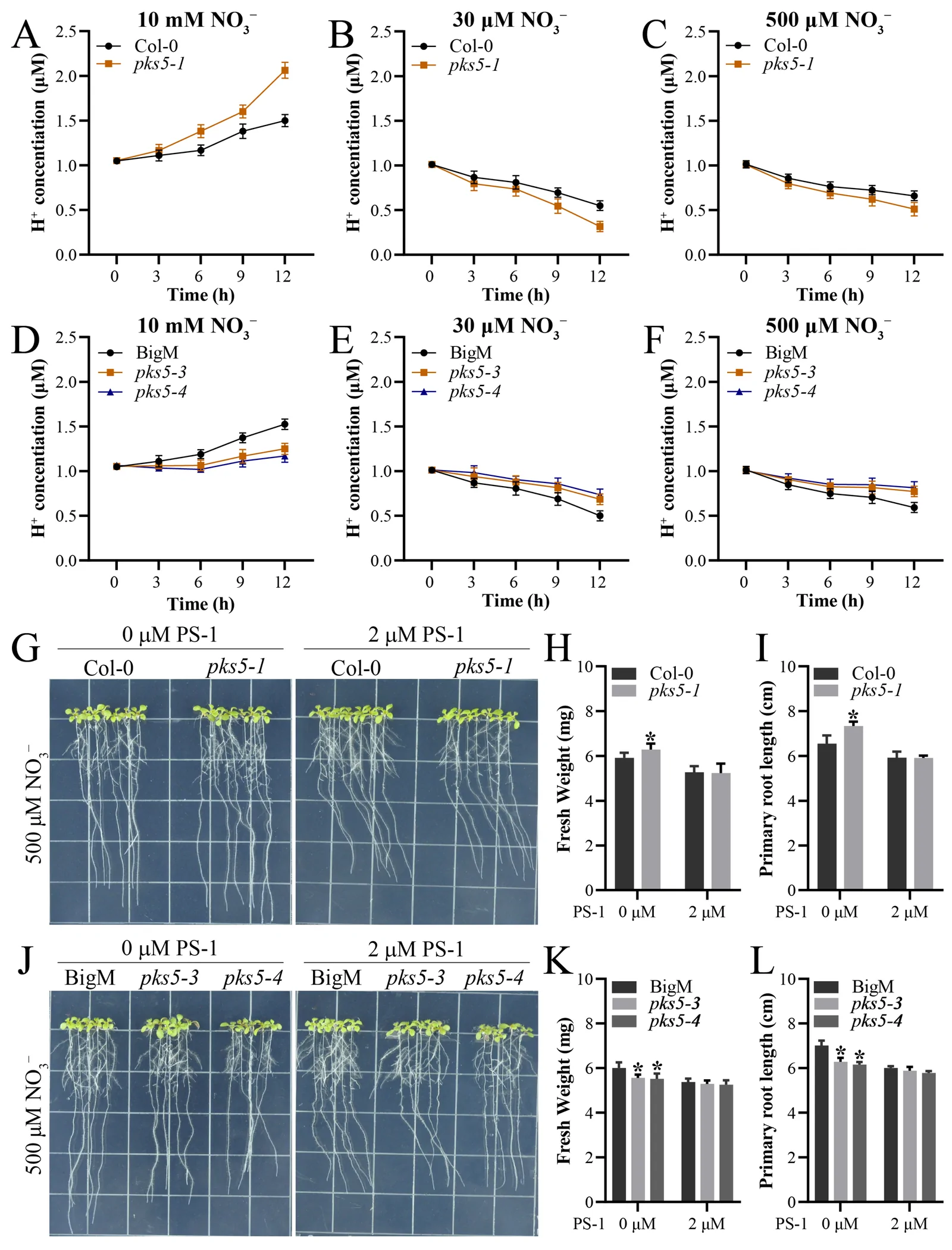
PM H^+^-ATPase participates in plant responses to changes in nitrate availability. (**A**–**C**) Extracellular H^+^ concentration in the culture medium of *pks5-1* and Col-0 seedlings under nitrate-sufficient (10 mM NO_3_^−^) and nitrate-deficient (30 μM and 500 μM NO_3_^−^) conditions. (**D**–**F**) Extracellular H^+^ concentration in the culture medium of *pks5-3*, *pks5-4*, and BigM seedlings under nitrate-sufficient (10 mM NO_3_^−^) and nitrate-deficient (30 μM and 500 μM NO_3_^−^) conditions. 14-day-old seedlings were transferred to liquid medium containing the indicated nitrate concentrations, with roots fully immersed. Extracellular H^+^ concentration was monitored over time using the bromocresol purple assay. (**G**) Growth phenotypes of Col-0 and *pks5-1* seedlings treated with the PM H^+^-ATPase inhibitor PS-1 under nitrate-deficient conditions. 5-day-old seedlings grown on MS medium containing 10 mM NO_3_^−^ were transferred to MS medium supplemented with 500 μM NO_3_^−^ in the presence of 2 μM PS-1. Representative images were taken after 5 days of treatment. (**H**) Fresh weight of seedlings shown in (**G**). (**I**) Primary root length of seedlings shown in (**G**). (**J**) Growth phenotypes of BigM, *pks5-3*, and *pks5-4* seedlings treated with PS-1 under nitrate-deficient conditions. Seedlings were treated as described in (**G**). (**K**) Fresh weight of seedlings shown in (**J**). (**L**) Primary root length of seedlings shown in (**J**). Data are presented as the mean ± SD of three biological replicates (*n* = 3). Statistical significance was determined using Student’s *t*-test for comparisons between Col-0 and *pks5-1*, and between BigM and *pks5-3* or *pks5-4* under the same treatment conditions; Asterisks indicate significant differences (*p* ≤ 0.05).

Because PM H^+^-ATPases drive proton extrusion and thereby promote extracellular acidification, extracellular H^+^ dynamics were monitored using the pH indicator bromocresol purple. Standard curves were established for each nitrate concentration (Figure S4A–I), allowing quantitative determination of extracellular H^+^ concentration. Under nitrate-sufficient conditions, extracellular H^+^ concentration increased over time in all genotypes (Figure 6A,D). Notably, the increase was more pronounced in the loss-of-function mutant *pks5-1* than in Col-0 after 12 h of nitrate-sufficient treatment (Figure S5A), whereas it was attenuated in the gain-of-function mutants *pks5-3* and *pks5-4* compared with BigM (Figure S5B), suggesting that CIPK11 negatively regulates extracellular acidification under nitrate-sufficient conditions. In contrast, under nitrate-deficient conditions (30 μM and 500 μM NO_3_^−^), extracellular H^+^ concentration decreased in all genotypes (Figure 6B,C,E,F). This decrease was greater in *pks5-1* but less pronounced in *pks5-3* and *pks5-4* relative to their respective controls after 12 h of nitrate-deficient treatment (Figure S5A,B), which may reflect enhanced proton consumption associated with nitrate uptake under nitrate-deficient conditions. Together with the corresponding changes in nitrate and protein accumulation, these findings suggest that CIPK11 modulates extracellular proton dynamics during nitrate responses. Given the established role of PM H^+^-ATPases in generating the proton motive force required for nitrate transport, CIPK11 may regulate nitrate acquisition and assimilation through modulation of PM H^+^-ATPase activity.

To further examine whether PM H^+^-ATPase activity contributes to the altered growth responses of the CIPK11 mutants, seedlings were treated with PS-1, a selective inhibitor of PM H^+^-ATPase activity [30]. Under nitrate-sufficient conditions, PS-1 had little effect on the growth differences between the mutants and their respective wild types (Figure S6A–F). Under nitrate-deficient conditions, however, PS-1 abolished the enhanced growth phenotype of *pks5-1*, resulting in growth comparable to that of Col-0 (Figure 6G–I). Likewise, PS-1 eliminated the growth differences between the gain-of-function mutants (*pks5-3* and *pks5-4*) and their corresponding wild type (BigM) (Figure 6J–L). Because PS-1 specifically inhibits PM H^+^-ATPase activity, these results suggest that the altered growth phenotypes of the CIPK11 mutants under nitrate-deficient conditions are largely dependent on PM H^+^-ATPase activity.

These results suggest that PM H^+^-ATPase activity is involved in CIPK11- regulated plant growth and physiological responses to nitrate availability.

## 3. Discussion

Nitrogen use efficiency (NUE) comprises nitrogen uptake efficiency and nitrogen utilization efficiency [31]. In this study, loss of function of CIPK11 enhanced plant adaptation to nitrate deficiency and was associated with changes in nitrate acquisition- and assimilation-related processes, as indicated by improved plant growth, increased expression of nitrate-responsive genes (*NRT2.1*, *NRT2.4*, *NIA1*, and *NIA2*), and higher nitrate and protein accumulation. Conversely, gain-of-function mutants of CIPK11 exhibited compromised adaptation to nitrate deficiency, accompanied by reduced nitrate and protein accumulation and altered expression of nitrate-responsive genes. These findings suggest that CIPK11 negatively affects NUE-related responses under nitrate-deficient conditions.

However, CIPK11 appeared to have limited effects on NUE-related traits under nitrate-sufficient conditions, based on growth performance and nitrate-responsive gene expression, despite its elevated transcript levels under these conditions. This raises an intriguing question regarding why CIPK11 is induced under nitrate sufficiency but repressed during nitrate deficiency. The transcriptional regulation of CIPK11 may be associated with its role in modulating plasma membrane H^+^-ATPase activity. CIPK11 has been reported to negatively regulate PM H^+^-ATPase activity through phosphorylation of AHA2 at Ser931 [26], although additional phosphorylation targets remain to be identified. PM H^+^-ATPase is a key regulator of nutrient acquisition and is tightly controlled to maintain appropriate activity under non-stress conditions because its activation requires substantial ATP consumption [20]. Plants coordinate nutrient acquisition with internal carbon and energy status through complex regulatory networks [7,32]. Our previous study showed that ATP-generating pathways, including glycolysis/gluconeogenesis, were activated under phosphate limitation but attenuated when sucrose availability was restricted [33]. Therefore, the repression of CIPK11 under nitrate deficiency may represent an adaptive mechanism that facilitates nutrient acquisition under energy-limited conditions, although its regulation may also be influenced by additional environmental and metabolic signals. Overall, downregulation of CIPK11 may contribute to enhanced NUE-related responses under nitrate-deficient conditions.

Transcriptional regulation is a major mechanism by which plants respond to changes in nitrate availability. The primary nitrate response following nitrate provision involves rapid transcriptional reprogramming of thousands of genes [34]. In addition to the Ca^2+^-CBL-CIPK module, the nitrate–Ca^2+^-dependent protein kinase (CPK)–NIN-like protein (NLP) signaling pathway plays a central role in regulating nitrate-responsive genes involved in transcriptional regulation, nitrate transport, and nitrogen assimilation [35,36]. Among the nine NLP family members in Arabidopsis, NLP6 and NLP7 are key regulators Nof nitrate-responsive gene expression, and in particular, CPK10/30/32-mediated phosphorylation of NLP7 at Ser205 promotes its nuclear retention and enhances NLP7-dependent transcriptional activation [36]. Moreover, NLP7 functions not only as a transcription factor but also as an intracellular nitrate sensor that directly links nitrate availability with downstream transcriptional responses [37]. Although primary nitrate responses occur within 15–30 min after nitrate provision [36,38], nitrate-responsive transcriptional changes can also be detected at later stages, such as 2–3 h after nitrate resupply [39,40,41].

In our study, time-course analysis showed that CIPK11 expression was gradually reduced under nitrate deficiency and reached its lowest level after 9 days of treatment. Therefore, a 3-day nitrate deficiency treatment was selected to examine nitrate-responsive gene expression. Following nitrate resupply, CIPK11 expression was strongly induced but remained lower than that under nitrate-sufficient conditions, and a 3 h resupply treatment was used for subsequent analyses. We found that CIPK11 modulated the expression of genes involved in nitrate uptake and assimilation, including nitrate transporter and nitrogen assimilation genes, which are also regulated by NLP-mediated nitrate signaling [35]. These findings suggest that CIPK11 may contribute to nitrate-responsive transcriptional regulation, either through interaction with established nitrate signaling pathways or via an independent regulatory mechanism. Whether nitrate-induced CIPK11 expression is directly controlled by NLP transcription factors remains to be determined.

Post-translational modifications, particularly protein phosphorylation and dephosphorylation, represent an additional layer of regulation in nitrate signaling and nutrient response adaptation [34,42]. For example, besides transcriptional regulation by factors such as LBD7/8/9 [43], the activity and stability of the high-affinity nitrate transporter NRT2.1 are dynamically controlled by phosphorylation events. Under nitrate-sufficient conditions, NRT2.1 activity is inhibited through phosphorylation of Ser501 by an unidentified kinase, whereas phosphorylation at Ser28 promotes NRT2.1 stabilization under nitrate limitation [43,44]. These findings highlight the importance of phosphorylation-mediated regulation in fine-tuning nitrate acquisition.

In addition to the Ca^2+^-CBL–CIPK and nitrate–CPK–NLP pathways, mitogen-activated protein kinase (MAPK) cascades have also emerged as important regulators of nitrate responses [11,36,38,42]. For example, MAPK6 regulates nitrogen assimilation by phosphorylating NIA, thereby affecting nitric oxide production in Arabidopsis [45]. Recently, an NLP-dependent MAP3K13/14–MKK3–MPK7 module was shown to contribute to nitrate responses during nitrate resupply after nitrogen starvation in Arabidopsis [38]. Moreover, MAP3Ks RAF14 and RAF79 in *Chlamydomonas reinhardtii* have been implicated in the regulation of nitrogen assimilation through ammonium-mediated repression, suggesting a conserved role of kinase signaling in nitrogen adaptation [42]. However, how distinct kinase networks, including CBL–CIPK, CPK–NLP, and MAPK cascades, interact to coordinate nitrate sensing, transport, and metabolism remains largely unresolved. Our findings provide additional evidence that CIPK-mediated signaling contributes to nitrate adaptation and suggest that CIPK11 may represent an important regulatory component linking Ca^2+^-dependent signaling with downstream nitrate response processes.

In the present study, nitrate availability strongly influenced extracellular proton status. Under nitrate-sufficient conditions, all genotypes exhibited extracellular acidification, with the *pks5-1* mutant showing enhanced H^+^ accumulation compared with the wild type, whereas the gain-of-function mutants *pks5-3* and *pks5-4* displayed reduced acidification. In contrast, nitrate deficiency decreased extracellular H^+^ levels in all genotypes, and this reduction was more pronounced in *pks5-1* but attenuated in *pks5-3* and *pks5-4*. These results indicate that CIPK11 modulates nitrate-dependent proton homeostasis. Under nitrate limitation, the lower extracellular H^+^ level observed in *pks5-1* may be associated with enhanced nitrate acquisition, in which increased activity of NO_3_^−^/H^+^ symporters could promote proton utilization for nitrate uptake. Consistently, the enhanced nitrate and protein accumulation in *pks5-1* suggests improved nitrogen acquisition and assimilation capacity. However, extracellular H^+^ concentration represents the net balance between proton extrusion, proton consumption, and buffering capacity, and therefore does not directly reflect PM H^+^-ATPase activity. Direct measurements of nitrate uptake using ^15^N labeling and proton flux using non-invasive techniques will be required to further verify the effects of CIPK11 on nitrate transport and proton dynamics.

Pharmacological inhibition of PM H^+^-ATPase activity using PS-1 partially influenced the growth responses of CIPK11 mutants under nitrate-deficient conditions. PS-1 has been reported to inhibit PM H^+^-ATPase activity by interacting with the central loop region of the proton pump and has been used to investigate biological processes involving PM H^+^-ATPase regulation [30]. Therefore, the differential sensitivity of CIPK11 mutants to PS-1 provides pharmacological evidence supporting the contribution of PM H^+^-ATPase-associated processes to CIPK11-mediated nitrate responses. Nevertheless, PS-1-induced growth alterations represent indirect consequences of PM H^+^-ATPase inhibition and cannot substitute for direct measurements of pump activity. PM H^+^-ATPases are primarily regulated through phosphorylation of their C-terminal autoinhibitory domains. Previous studies demonstrated that CIPK11 phosphorylates AHA2 at Ser931 under nitrate-sufficient conditions, thereby maintaining PM H^+^-ATPase activity at a relatively low level. In this context, nitrate deficiency-induced downregulation of CIPK11 expression may alleviate this inhibitory regulation, potentially promoting PM H^+^-ATPase activation and facilitating nitrate adaptation. Whether CIPK11 regulates additional phosphorylation sites, including the activation-associated Thr947 residue of AHA2, and whether these phosphorylation events directly contribute to nitrate uptake efficiency remain important questions for future investigation.

**Figure 7:**
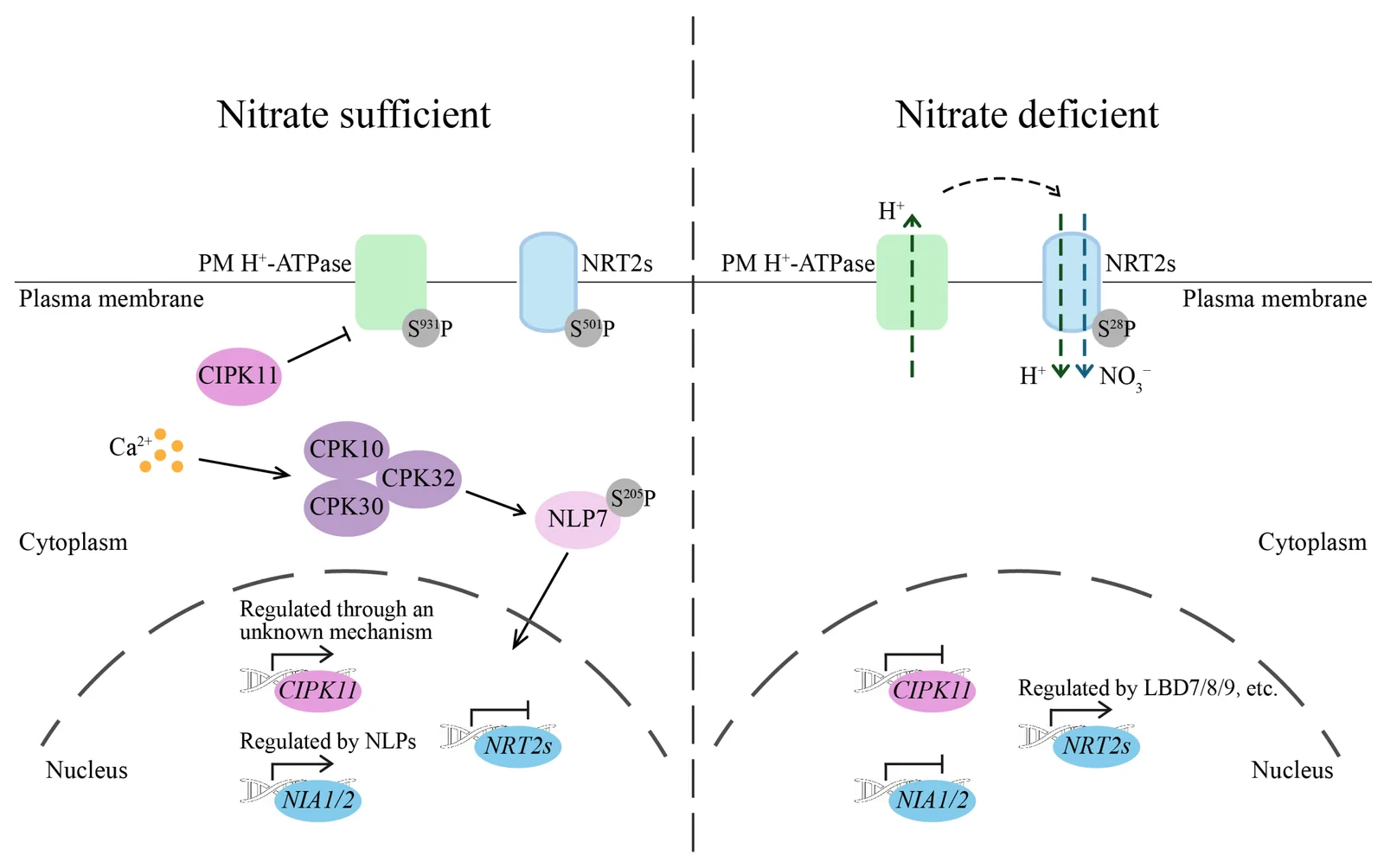
Proposed working model for CIPK11-mediated regulation of plant responses to nitrate availability. Under nitrate-sufficient conditions, CIPK11 expression is maintained at a relatively high level through an unknown mechanism and negatively regulates plasma membrane H^+^-ATPase activity through phosphorylation on Ser931 of AHA2 based on previous reports [26], thereby limiting proton extrusion. Meanwhile, *NRT2s* remain relatively lowly expressed and is phosphorylated at Ser501 on NRT2.1 to inhibit its activity under sufficient nitrate conditions. Nitrate-induced calcium signaling promotes CPK10/30/32-mediated phosphorylation and nuclear localization of NLP7 at Ser205, which activates nitrate assimilation genes, including *NIA1/2*. Under nitrate-deficient conditions, CIPK11 expression is reduced, potentially relieving its inhibitory effect on plasma membrane H^+^-ATPase activity and enhancing H^+^ extrusion to facilitate nitrate uptake through NRT2s. Nitrate deficiency induces *NRT2s* expression and phosphorylated at Ser28 on NRT2.1 to promotes its stabilization, whereas *NIA1/2* expression is reduced. Altered CIPK11 activity can modulates the expression responses of *NRT2s* and *NIA1/2* with nitrate availability through unknown mechanism. Solid arrows indicate relationships supported by experimental evidence from this study or previous reports, whereas dashed arrows indicate proposed mechanisms that remain to be validated.

Taken together, our findings support a model in which CIPK11 acts as an important regulator of plasma membrane H^+^-ATPase-associated processes during plant adaptation to changing nitrate availability (Figure 7). Under nitrate-sufficient conditions, CIPK11 may contribute to maintaining relatively low PM H^+^-ATPase activity through phosphorylation-dependent regulation of AHA2, consistent with previous reports showing that CIPK11 negatively regulates AHA2 activity via phosphorylation of Ser931 [26]. Under nitrate-deficient conditions, reduced CIPK11 expression may alleviate this inhibitory regulation, potentially promoting PM H^+^-ATPase activation and facilitating the establishment of proton gradients required for nitrate uptake. The dynamic regulation of proton homeostasis may subsequently support NO_3_^−^/H^+^ symport-mediated nitrate acquisition and nitrogen adaptation. Further investigation of the upstream signals controlling *CIPK11* expression and the downstream regulatory mechanisms will provide deeper insights into how plants balance nitrogen acquisition and utilization under changing environmental conditions.

## 4. Materials and Methods

### 4.1. Plant Materials and Growth Conditions

*Arabidopsis thaliana* ecotypes Columbia-0 (Col-0) and Columbia erecta105 (BigM) were used as the wild-type controls in this study. The CIPK11 loss-of-function mutant *pks5-1* (SALK_108074), carrying a kinase-impaired allele, is in the Col-0 background. The gain-of-function mutants *pks5-3* (CS90635) and *pks5-4* (CS91114), which carry constitutively active CIPK11 alleles containing the S317L and A168V substitutions, respectively, are in the BigM background. All mutant lines were kindly provided by Prof. Yan Guo (China Agricultural University, Beijing, China), and their genetic characterization has been described previously [26,46,47]. The significantly reduced expression of *CIPK11* in *pks5-1* seedlings was verified by qRT-PCR (Figure S7).

Murashige and Skoog (MS) medium containing only 10 mM NO_3_^−^ as the nitrogen source was used as the nitrate-sufficient medium. Nitrate-deficient media were prepared by replacing KNO_3_ with an equimolar concentration of KCl while maintaining the concentrations of all other nutrients. Media were supplemented with 2% (*w*/*v*) sucrose, adjusted to pH 5.8, and solidified with 0.5% (*w*/*v*) Phytagel for vertical culture. The reagents used in this study were ordered from Yuanye Biotechnology (Shanghai, China).

Seeds were surface-sterilized in 8% (*v*/*v*) sodium hypochlorite containing 0.2% (*v*/*v*) Triton X-100 for 15 min, rinsed three times with sterile distilled water, and sown on the indicated media. After stratification at 4 °C for 2 days, seedlings were grown in a controlled-environment growth chamber (Right Science, Hefei, China) at 22 °C under a 16 h light/8 h dark photoperiod with a light intensity of 100 μmol m^−2^ s^−1^.

For nitrate deprivation treatment, 5-day-old seedlings grown vertically on nitrate-sufficient MS medium were transferred to media containing the indicated nitrate concentrations and cultured for the times specified in each experiment. For nitrate-resupply treatment, seedlings were first transferred to nitrate-deficient medium for 3 days and subsequently returned to nitrate-sufficient medium for the indicated durations before sample collection. Lateral root length was defined as the average length of emerged first-order lateral roots longer than 1 mm per seedling. The number of emerged first-order lateral roots longer than 1 mm was counted for each seedling. Lateral root density was calculated as the number of emerged first-order lateral roots per unit length of the primary root. Root length and lateral root number were measured using Image J software (version 1.37v, Wayne Rasband, National Institutes of Health, USA).

### 4.2. Nitrate Content Determination

Nitrate content was determined according to previously described methods [48] with minor modifications. Briefly, seedlings (≤100 mg fresh weight per sample) were collected into 1.5 mL of microcentrifuge tubes containing 2.8 mm steel beads, immediately frozen in liquid nitrogen, and homogenized under liquid nitrogen. The powdered samples were extracted with 800 μL of deionized water at 45 °C for 1 h, followed by centrifugation at 12,000× *g* for 10 min at room temperature. An aliquot (10 µL) of the supernatant was mixed with 40 µL of 5% (*w*/*v*) salicylic acid in sulfuric acid and incubated at room temperature in the dark for 20 min. Subsequently, 700 μL of 9.5% (*w*/*v*) NaOH was added, and the mixture was thoroughly mixed and allowed to cool to room temperature. The absorbance was measured at 410 nm using a microplate reader. Nitrate content was calculated based on a KNO_3_ standard curve. The residual nitrate derived from other components during medium preparation was measured and defined as the background nitrate level. The background nitrate concentration was approximately 30 μM, which was used as the severe nitrate-deficient condition in this study.

### 4.3. Anthocyanin Content Determination

Anthocyanin content was determined as previously reported [33]. Briefly, seedlings subjected to different nitrate treatments (≤100 mg fresh weight per sample) were collected into 1.5 mL microcentrifuge tubes containing 2.8 mm steel beads, immediately frozen in liquid nitrogen, and homogenized under liquid nitrogen. Anthocyanin was extracted with 500 µL of 1% (*v*/*v*) HCl in ethanol using ultrasonic treatment for 20 min. The extracts were then centrifuged at 12,000× *g* for 10 min at 4 °C, and the supernatants were collected for analysis. Absorbance was measured at 530 nm using a microplate reader, and anthocyanin content was calculated based on a commercial anthocyanin standard curve.

### 4.4. Soluble Protein Content Determination

Soluble protein content was determined using a commercial protein assay kit (Yuanye Biotechnology, Shanghai, China) according to the manufacturer’s instructions. Briefly, seedlings (≤100 mg fresh weight per sample) were collected into 1.5 mL of microcentrifuge tubes containing 2.8 mm steel beads, immediately frozen in liquid nitrogen, and homogenized under liquid nitrogen. The powdered samples were extracted with 800 μL of protein extraction buffer (10 mM Tris-HCl, 150 mM NaCl, 2 mM EDTA, 0.5% (*v*/*v*) NP-40, pH 7.6) at 4 °C for 2 h with gentle rotation. The extracts were then centrifuged at 12,000× *g* for 10 min at 4 °C, and the resulting supernatants were collected. Soluble protein content was subsequently quantified using the aforementioned kit following the manufacturer’s protocol.

### 4.5. Extracellular Acidification Assay

For quantitative determination of extracellular H^+^ concentration, a standard curve was established with minor modifications based on a previously described method [49]. Briefly, separate standard curves were generated using MS media containing different nitrate concentrations to minimize the influence of nitrate on pH stability. The pH of each medium was adjusted to values ranging from 5.4 to 6.6. An aliquot of 198 μL of each pH standard solution was mixed with 2 μL of bromocresol purple stock solution (0.6%, *w*/*v*, dissolved in ethanol) to a final concentration of 0.006% (*w*/*v*). Absorbance was measured at 434 nm and 588 nm using a microplate reader, and standard curves were generated based on the absorbance ratio (A_434_/A_588_).

For extracellular acidification measurements, 14-day-old seedlings grown vertically on solid MS medium were gently rinsed with deionized water and immediately transferred to liquid MS medium containing the indicated nitrate concentrations. At 0, 3, 6, 9, and 12 h after transfer, aliquots of the culture medium were collected and mixed with bromocresol purple stock solution (0.6%, *w*/*v*) to a final concentration of 0.006% (*w*/*v*). Absorbance was measured at 434 nm and 588 nm using a microplate reader, and extracellular H^+^ concentration was calculated according to the corresponding standard curve.

### 4.6. PS-1 Treatment

PS-1 was kindly provided by Prof. Yongqing Yang (China Agricultural University, Beijing, China). A 20 mM PS-1 stock solution was prepared in dimethyl sulfoxide (DMSO). After sterilization of the culture medium, PS-1 was added to the medium to a final concentration of 2 μM. Control medium received the same volume of DMSO as the solvent control.

### 4.7. Quantitative Real-Time PCR (qRT-PCR) Analysis

Total RNA was extracted from seedlings using the RNAprep Pure Plant Kit (DP432, TIANGEN, Beijing, China) according to the manufacturer’s instructions. RNA quality and concentration were assessed using a NanoDrop spectrophotometer (ASP2680, ACTGene, Piscataway, NJ, USA). cDNA synthesis was performed using the PrimeScript RT reagent Kit with gDNA Eraser (RR047A, TaKaRa, Shiga, Japan) according to the manufacturer’s instructions. qRT-PCR was performed using TB Green Premix Ex kit (RR420A, TaKaRa, Shiga, Japan) on a LightCycler 480 II instrument (Roche, Mannheim, Germany). *ACTIN* was used as the internal control. Primers used in this study are listed in Table S1.

### 4.8. Quantification and Statistical Analysis

Root length was measured using Image J software (version 1.37v, Wayne Rasband, National Institutes of Health, USA). Statistical analyses were performed using Graphpad Prism 8 software. Differences between two groups were evaluated using a two-tailed unpaired Student’s *t*-test. All experiments were independently repeated at least three times with similar results. Data are presented as the mean ± SD unless otherwise indicated. A *p* value ≤ 0.05 was considered statistically significant.

## 5. Conclusions

Overall, our study establishes CIPK11 as a previously unrecognized component of the nitrate signaling network that negatively regulates plant adaptation to nitrate deficiency. We demonstrate that CIPK11 modulates plant responses to limited nitrate availability through coordinated regulation of nitrate-responsive gene expression, plasma membrane H^+^-ATPase activity, and nitrate acquisition and assimilation processes. These findings reveal an important role of CIPK11 in integrating nitrate signaling with physiological responses and expand our understanding of CIPK-mediated regulation of nitrogen adaptation in plants.

## Data Availability

The original contributions presented in this study are included in the article. Further inquiries can be directed to the corresponding author(s).

## Supplementary Materials

The following supporting information can be downloaded at: https://www.mdpi.com/article/10.3390/plants15172723/s1, Figure S1: Relative expression levels of *CIPK11* and nitrate-responsive genes under nitrate deprivation followed by nitrate resupply compared with continuous nitrate provision; Figure S2: CIPK11 regulates nitrate and protein accumulation under nitrate-deficient conditions (500 μM NO_3_^−^); Figure S3: CIPK11 regulates net protein accumulation during nitrate recovery; Figure S4: Standard curves for extracellular H^+^ determination using bromocresol purple; Figure S5: Quantification of extracellular H^+^ concentration after 12 h of nitrate-sufficient and nitrate-deficient treatment; Figure S6: Effects of PS-1 on seedling growth under nitrate-sufficient condition; Figure S7: qRT-PCR analysis of *CIPK11* expression in the *pks5-1* mutant. Table S1: Primers used in this study.
