# Supplementary material for: CIPK11 Regulates Plant Adaptation to Nitrate Availability in Arabidopsis
Source: Plants (Basel). 2026 Sep 5;15(17):2723. doi: 10.3390/plants15172723 (PMC13567909; doi:10.3390/plants15172723)
Supplement: Supplementary file 1 [file plants-15-02723-s001.zip › plants-4469529-supplementary.pdf]

CIPK11 regulates plant adaptation to nitrate availability in Arabidopsis

Supplemental Figures and Table

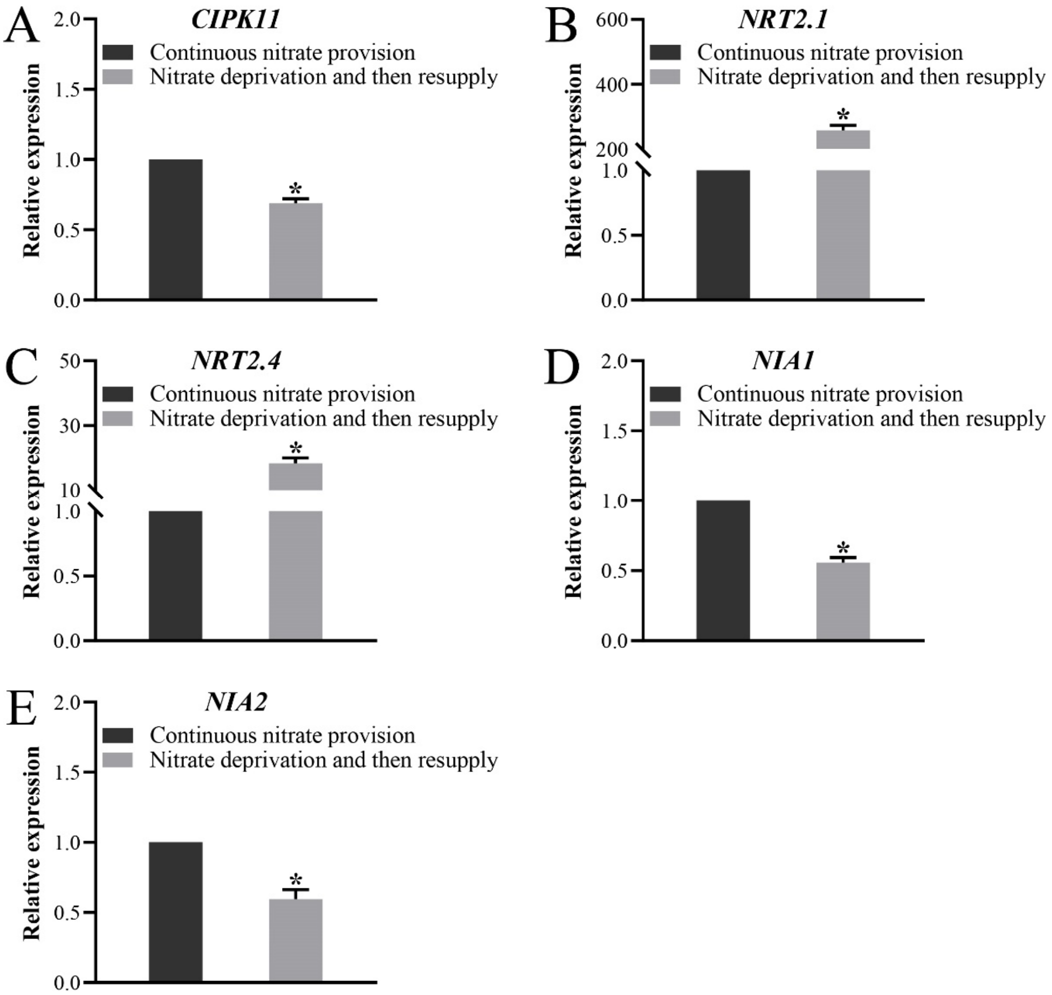

Figure S1. Relative expression levels of *CIPK11* and nitrate-responsive genes under nitrate deprivation followed by nitrate resupply compared with continuous nitrate provision. (**A**) Relative expression level of *CIPK11*. (**B–E**) Relative expression levels of nitrate-responsive genes, including *NRT2.1*, *NRT2.4*, *NIA1*, and *NIA2*. 5-day-old seedlings grown on MS medium containing sufficient nitrate (10 mM NO_3^-) were divided into two groups. One group was transferred to nitrate-deficient medium (30 μM NO_3^-) for 3 days and subsequently transferred back to nitrate-sufficient medium (10 mM NO_3^-) for 3 h, whereas the other group was transferred to nitrate-sufficient medium (10 mM NO_3^-) as the control. Seedlings subjected to nitrate deprivation followed by resupply and continuous nitrate provision were harvested for RNA extraction and gene expression analysis. Data are presented as the mean ± SD of three biological replicates (n = 3). Statistical significance was determined using Student's t-test for comparisons between nitrate deprivation followed by resupply and continuous nitrate provision; Asterisks indicate significant differences ($P \leq 0.05$).

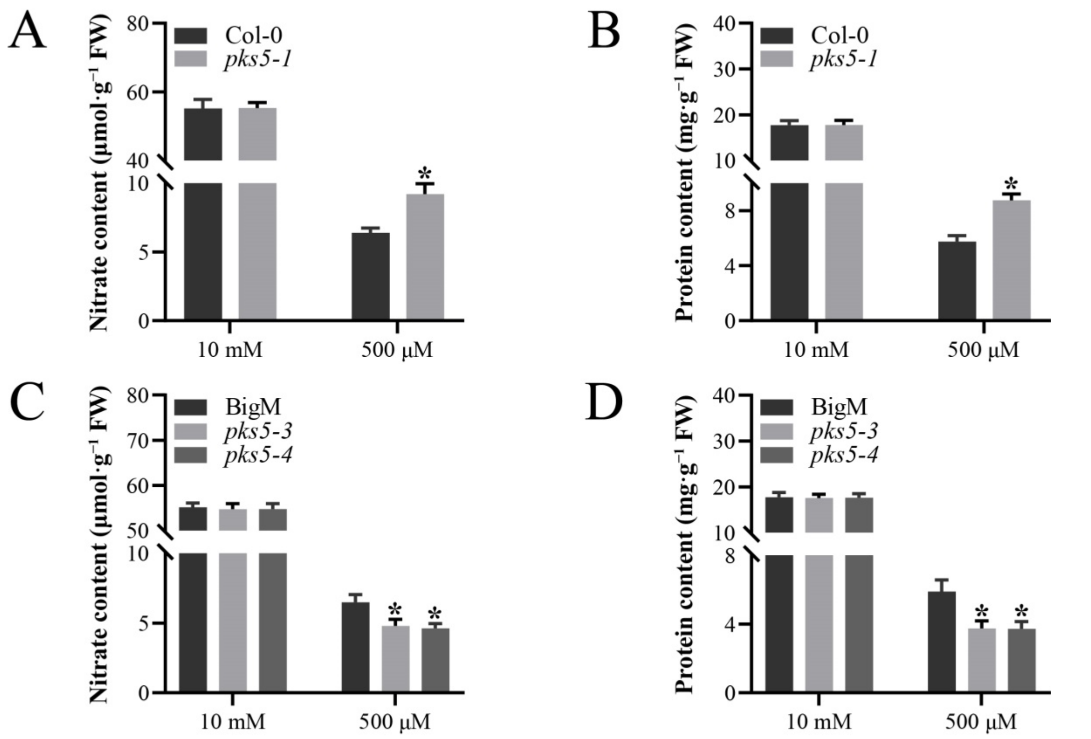

Figure S2. CIPK11 regulates nitrate and protein accumulation under nitrate-deficient conditions (500 μM NO_3^-). (**A**) Nitrate content in Col-0 and *pks5-1* seedlings. (**B**) Protein content in Col-0 and *pks5-1* seedlings. (**C**) Nitrate content in BigM, *pks5-3* and *pks5-4* seedlings. (**D**) Protein content in BigM, *pks5-3* and *pks5-4* seedlings. 5-day-old seedlings grown on MS medium containing sufficient nitrate (10 mM NO_3^-) were transferred to nitrate-deficient medium containing 500 μM NO_3^- for 3 days. Seedlings were then harvested for nitrate and protein content analyses. FW, fresh weight. Data are presented as the mean ± SD of three biological replicates (n = 3). Statistical significance was determined using Student's t-test for comparisons between Col-0 and *pks5-1*, and between BigM and *pks5-3* or *pks5-4* under the same treatment condition; Asterisks indicate significant differences ($P \leq 0.05$).

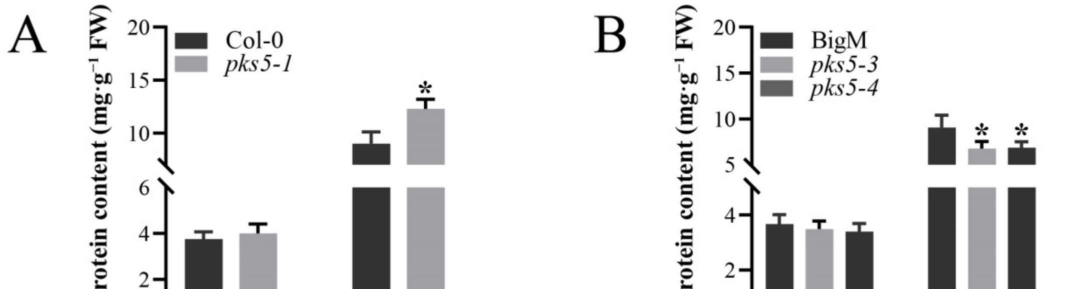

Figure S3. CIPK11 regulates net protein accumulation during nitrate recovery. (**A**) Net protein content after nitrate resupply in Col-0 and *pks5-1* seedlings. (**B**) Net protein content after nitrate resupply in BigM, *pks5-3* and *pks5-4* seedlings. 5-day-old seedlings grown on MS medium containing sufficient nitrate (10 mM NO_3^-) were transferred to nitrate-deficient medium (30 μM NO_3^-) for 3 days. For nitrate-resupply treatment, seedlings were subsequently transferred to nitrate-sufficient medium (10 mM NO_3^-) and harvested at the indicated time points for protein content analyses. Net protein accumulation was calculated as the difference between protein content after nitrate resupply and that before resupply. FW, fresh weight. Data are presented as the mean ± SD of three biological replicates (n = 3). Statistical significance was determined using Student's t-test for comparisons between Col-0 and

pks5-1, and between BigM and *pks5-3* or *pks5-4* under the same treatment condition; Asterisks indicate significant differences ($P \leq 0.05$).

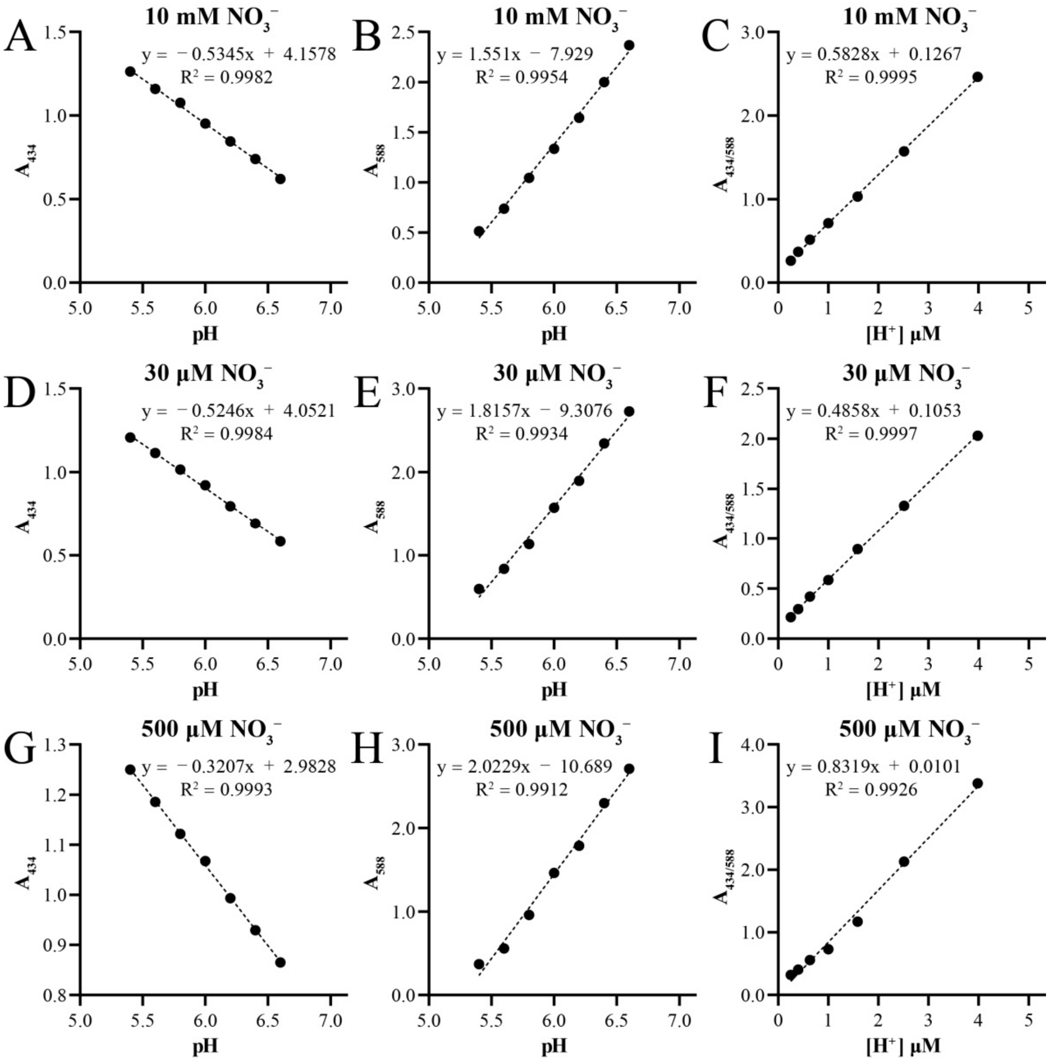

Figure S4. Standard curves for extracellular H^+ determination using bromocresol purple. (**A**) Absorbance of bromocresol purple at 434 nm at different pH values in MS medium containing 10 mM NO_3^-. (**B**) Absorbance of bromocresol purple at 588 nm at different pH values in MS medium containing 10 mM NO_3^-. (**C**) Standard curve showing the relationship between the A_{434}/A_{588} ratio and H^+ concentration in MS medium containing 10 mM NO_3^-. (**D**) Absorbance of bromocresol purple at 434 nm at different pH values in MS medium containing 30 μM NO_3^-. (**E**) Absorbance of bromocresol purple at 588 nm at different pH values in MS medium containing 30 μM NO_3^-. (**F**) Standard curve showing the relationship between the A_{434}/A_{588} ratio and H^+ concentration in MS medium containing 30 μM NO_3^-. (**G**) Absorbance of bromocresol purple at 434 nm at different pH values in MS medium containing 500 μM NO_3^-. (**H**) Absorbance of bromocresol purple at 588 nm at different pH values in MS medium containing 500 μM NO_3^-. (**I**) Standard curve showing the relationship between the A_{434}/A_{588} ratio and H^+ concentration in MS medium containing 500 μM NO_3^-.

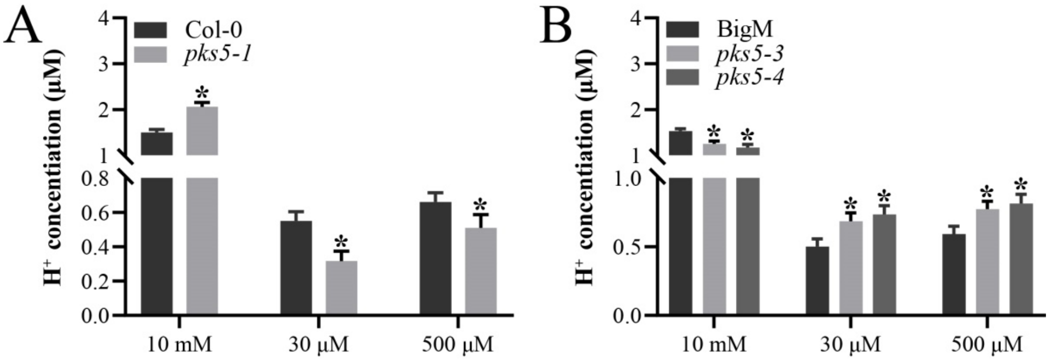

Figure S5. Quantification of extracellular H^+ concentration after 12 h of nitrate-sufficient and nitrate-deficient treatment. (**A**) Extracellular H^+ concentration in the culture medium of Col-0 and *pks5-1* seedlings after 12 h of nitrate-sufficient and nitrate-deficient treatment. (**B**) Extracellular H^+ concentration in the culture medium of BigM, *pks5-3* and *pks5-4* seedlings after 12 h of nitrate-sufficient and nitrate-deficient treatment. 14-day-old seedlings were transferred to liquid medium containing the indicated nitrate concentrations. Extracellular H^+ concentration was determined using the bromocresol purple assay. Data are presented as the mean ± SD of three biological replicates (n = 3). Statistical significance was determined using Student's *t*-test for comparisons between Col-0 and *pks5-1*, and between BigM and *pks5-3* or *pks5-4* under the same treatment condition; Asterisks indicate significant differences ($P \leq 0.05$).

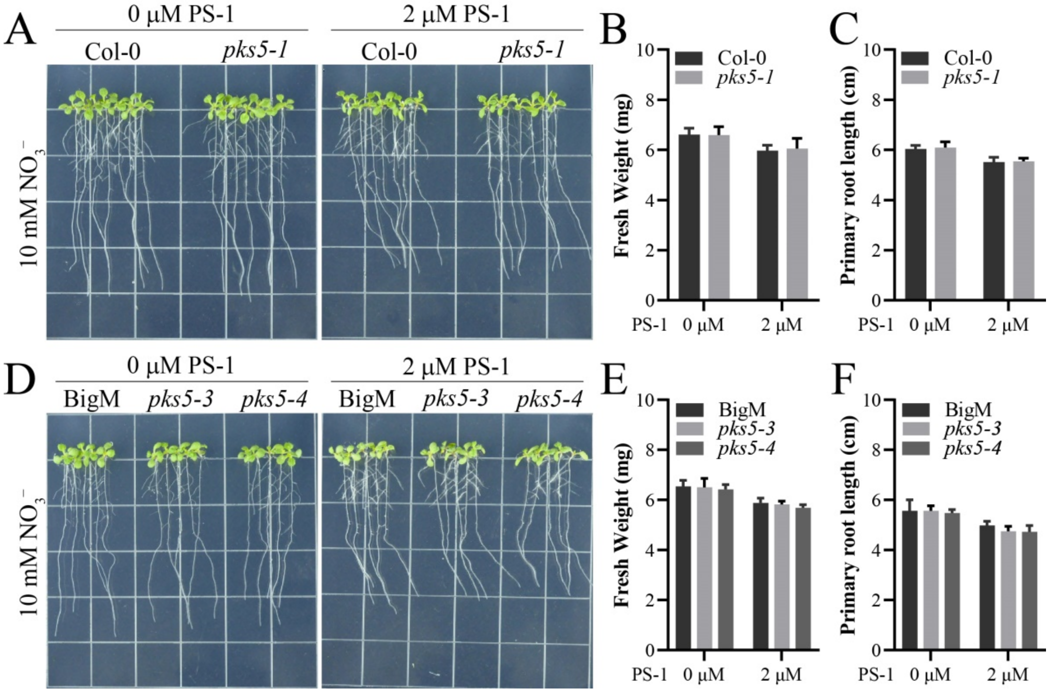

Figure S6. Effects of PS-1 on seedling growth under nitrate-sufficient condition. (**A**) Growth phenotypes of Col-0 and *pks5-1* seedlings treated with the PM H^+-ATPase inhibitor PS-1 under nitrate-sufficient conditions. (**B**) Fresh weight of seedlings shown in (**A**). (**C**) Primary root length of seedlings shown in (**A**). (**D**) Growth phenotypes of BigM, *pks5-3*, and *pks5-4* seedlings treated with PS-1 under nitrate-sufficient conditions. (**E**) Fresh weight of seedlings shown in (**D**). (**F**) Primary root length of seedlings shown in (**D**). 5-day-old seedlings grown on MS medium containing 10 mM NO_3^- were transferred to MS medium containing 10 mM NO_3^- with or without 2 μM PS-1. Representative images were taken after 5 days of treatment. Data are presented as the mean ± SD of three biological replicates (n

= 3). Statistical significance was determined using Student's *t*-test for comparisons between Col-0 and *pks5-1*, and between BigM and *pks5-3* or *pks5-4* under the same treatment condition.

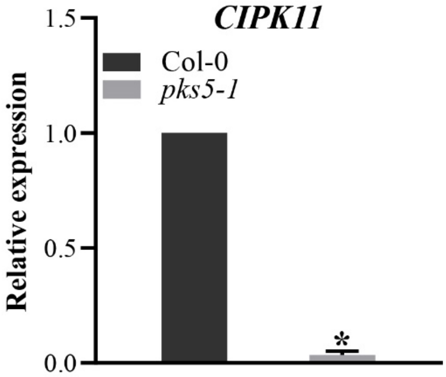

Figure S7. qRT-PCR analysis of *CIPK11* expression in the *pks5-1* mutant. Data are presented as the mean ± SD of three biological replicates (n = 3). Statistical significance was determined using Student's *t*-test for comparisons between Col-0 and *pks5-1*; Asterisks indicate significant differences ($P \leq 0.05$).

Table S1. Primers used in this study.

Primer	Sequence (5′-3′)
CIPK11-F	GAAGGTGCTAAAGTTGATGTATGGTCT
CIPK11-R	CGTCATCGTGGAACTTGATCTGTTT
NRT2.1-F	AACAAGGGCTAACGTGGATG
NRT2.1-R	CTGCTTCTCCTGCTCATTCC
NRT2.4-F	GAACAAGGGCTGACATGGAT
NRT2.4-R	GCTTCTCGGTCTCTGTCCAC
NIA1-F	GTGATCCAGAGGACGAAACC
NIA1-R	AACCTTCCTTTGCGATTTCA
NIA2-F	TCACGAGGAGATTCTTCCCA
NIA2-R	CTTCCCATACTTGTTTGGC
ACTIN-F	GGTAACATTGTGCTCAGTGGTGG
ACTIN-R	AACGACCTTAATCTTCATGCTGC
